# The clinical potential of radiomics to predict hematoma expansion in spontaneous intracerebral hemorrhage: a narrative review

**DOI:** 10.3389/fneur.2024.1427555

**Published:** 2024-07-19

**Authors:** Samuel A. Tenhoeve, Matthew C. Findlay, Kyril L. Cole, Diwas Gautam, Jayson R. Nelson, Julian Brown, Cody J. Orton, Michael T. Bounajem, Michael G. Brandel, William T. Couldwell, Robert C. Rennert

**Affiliations:** ^1^Spencer Fox Eccles School of Medicine, University of Utah, Salt Lake City, UT, United States; ^2^Department of Neurosurgery, Clinical Neurosciences Center, University of Utah, Salt Lake City, UT, United States; ^3^Department of Neurosurgery, University of California San Diego, San Diego, CA, United States

**Keywords:** radiomics, machine learning, spontaneous intracerebral hemorrhage, hematoma expansion, noncontrast CT imaging

## Abstract

Spontaneous intracerebral hemorrhage (sICH) is associated with significant morbidity and mortality, with subsequent hematoma expansion (HE) linked to worse neurologic outcomes. Accurate, real-time predictions of the risk of HE could enable tailoring management—including blood pressure control or surgery—based on individual patient risk. Although multiple radiographic markers of HE have been proposed based on standard imaging, their clinical utility remains limited by a reliance on subjective interpretation of often ambiguous findings and a poor overall predictive power. Radiomics refers to the quantitative analysis of medical images that can be combined with machine-learning algorithms to identify predictive features for a chosen clinical outcome with a granularity beyond human limitations. Emerging data have supported the potential utility of radiomics in the prediction of HE after sICH. In this review, we discuss the current clinical management of sICH, the impact of HE and standard imaging predictors, and finally, the current data and potential future role of radiomics in HE prediction and management of patients with sICH.

## 1 Introduction

Spontaneous intracerebral hemorrhage (sICH) refers to acute bleeding in the absence of trauma within the brain parenchyma that can expand into the ventricular, subarachnoid, and subdural spaces (1). The primary and most common causes of sICH are hypertension or underlying amyloid angiopathy; secondary causes of sICH include acquired or congenital coagulopathies, drugs, and underlying tumors or vascular lesions (2). sICH results in significant morbidity and mortality, accounting for 10–15% of all hemorrhagic strokes (3), which in turn have a global incidence and mortality of over 5 million and 3 million people, respectively (3).

Perhaps the most devastating feature of sICH is the propensity for hematoma expansion (HE), which occurs in up to one third of patients (4) and is a significant predictor of mortality and poor functional outcomes (5). Nearly 40% of HE occurs within three hours of symptom onset (6), and its occurrence often precipitates neurologic deterioration as indicated by a decline in Glasgow Coma Scale (GCS) score. Identifying patients at risk and limiting HE in those with initially smaller hematoma volumes and higher GCS scores is particularly important to optimize outcomes, given that patients in one study with an overall hematoma volume of >60 mL and GCS score ≤ 8 had a 90% mortality rate at one month after initial presentation (7).

Although several clinical risk factors have been identified to help predict the likelihood of ICH formation (e.g., hypertension, genetic disposition, and cerebral amyloid angiopathy), the predictability of HE is less clear (8). Current imaging techniques such as specialized computed tomography (CT) and magnetic resonance imaging (MRI) scans are used to confirm the diagnosis of ICH (9), but predictions of HE risk often rely on human detection of gross imaging anomalies such as active extravasation. Radiomics, an emerging field using quantitative data extraction from diagnostic images for clinical problem-solving, represents a potential tool to address this issue (10).

Specifically, radiomics integrates numerous images, image derivatives, and higher-order data mined from medical images to identify complex patterns not otherwise visible to aid in pathology identification and management (11). Because of its quantitative and integrative nature, radiomics has the potential to significantly improve patient care beyond the limits of human ability. In one recent study, a radiomics-based model outperformed the diagnostic expertise of four experienced radiologists by identifying separate tumor pathologies (renal cell carcinoma, fat-poor angioleiomyolipoma, and oncocytoma) at a median sensitivity of 90.0, 86.3, and 73.4%, with a specificity of 89.1, 83.3, and 91.3%, respectively (12). In comparison, the four radiologists performed at a median sensitivity of 84.5% for each tumor type and a median specificity of 61.8, 80.6, and 50.0%. Accordingly, radiomics is emerging as a potential tool to predict HE in patients with sICH (13). In the present review, we examine the current status and future of radiomics in HE detection and sICH management.

## 2 Methods

The search for relevant literature was performed using the electronic database Pubmed^1^ and included iterations of the search terms “spontaneous intracerebral hemorrhage,” “intraparenchymal hemorrhage,” “hematoma expansion,” “predict,” “imaging,” and “radiomics.” Relevant articles identified on manual review are summarized in the manuscript text, with specific information extracted for the summary tables including author; publication and data collection year(s); study design and number of included studies; total number of patients; inclusion and exclusion criteria; imaging modality and features of focus; main study findings; limitations, and/or potential sources of bias (Tables 1, 2).

**Table 1:** Summary of clinical trials and pilot analyses evaluating manual imaging methodologies for predicting hematoma expansion in spontaneous intracerebral hemorrhage.

Study authors, date	(1) Study design; (2) N studies; (3) N patients; (4) years studied	(1) Inclusion criteria; (2) exclusion criteria	(1) Primary outcome; (2) secondary outcome	Imaging modality and radiology signs	Main study findings	Limitations and interpretation
Kuohn et al. 2022 (14)	Post-hoc analysis of RCT patients from FAST trial 1 728 2000–2010	>18 years old, spontaneous ICH confirmed on brain CT within 3 h of onset Admission GCS <5, surgical ICH evacuation planned within 24 h of admission, a known or suspected secondary cause of ICH, prior disability (mRS score > 2), or known thrombotic or vaso-occlusive disease within 30 days of ICH onset	HE (increase of >33% or > 6 mL) Early neurological deterioration, mRS at 90 days	Standard NCCT; ICH location	Lobar ICH location is associated with larger ICH volume, more HE, and early neurologic deterioration. Deep ICH is associated with worse outcomes.	Several studies (41, 42) have demonstrated that deep ICH, rather than lobar, is at higher risk of HE. However, location alone is a poor prognostic indicator for HE.
Brouwers et al. 2014 (15)	Prospective cohort 2 centers 1,012 2010–2012 & 1994–2011	Diagnosis of primary ICH, baseline, and follow-up CT available for volume analysis Patient had undergone hematoma evacuation before follow-up CT, intraventricular hemorrhage, or patient had a known or suspected secondary cause of ICH	HE (>6 mL or 33% growth)	Standard NCCT; ICH initial size	A 9-point prediction score for HE was developed and independently validated; it included initial ICH size as an important predictor.	Although the 9-point prediction score developed and validated herein demonstrates utility in predicting HE, initial ICH size is only one variable and is not sufficient to predict HE alone. However, when included as part of this 9-point prediction score, it may provide potential benefit in predicting HE.
Yu et al. 2017 (16)	Systemic review and meta-analysis 7 2,294 N/A	Studies including the following combinations: CT, NCCT, shape, density, hypodensity, blend sign, black hole sign, swirl sign, and intracerebral hemorrhage or ICH Outcomes other than HE, lack of essential data, duplicate data	Pooled sensitivity, specificity, positive likelihood ration, negative likelihood ratio of predicting HE by: Shape irregularity of ICH Density irregularity of ICH	Standard NCCT; ICH shape irregularities	Irregular ICH shape on NCCT has an overall sensitivity, specificity, and AUC of 67, 47%, and 0.61, respectively, for predicting HE.	The predictive value of shape irregularity and density heterogeneity of a hematoma for HE is limited, given the relatively low sensitivity, specificity, and AUC. Better NCCT predictors for HE in sICH may exist.
Yu et al. 2017 (17)	Meta-analysis 5 2,248 N/A	Original studies about predictive accuracy of blend sign for HE in ICH Duplication, non-related titles and abstracts, no clear time of initial CT scan, or no mention of blend sign	Pooled sensitivity, specificity, and SROC curve of predicting HE by: Blend sign for HE prediction	Standard NCCT; blend sign	Blend sign is a useful predictor with high specificity for HE in ICH. Sensitivity, specificity, and AUC of the blend sign for HE were 28, 92%, and 0.85, respectively.	The predictive value of blend sign alone demonstrates relatively high specificity for HE prediction. However, limitations in sensitivity and AUC prevent its reliable use alone.
Zheng et al. 2018 (18)	Meta-analysis 5 1,495 N/A	Original studies on the association between the black hole sign and HE in ICH Duplication, non-related titles and abstracts, no clear time of initial CT scan, or no mention of the black hole sign	Pooled sensitivity, specificity, and SROC curve of predicting HE by: Black hole sign for HE prediction	Standard NCCT; black hole sign	The black hole sign is a helpful imaging marker for predicting HE in ICH. Sensitivity, specificity, and AUC of 30, 91%, and 0.78, respectively.	The black hole sign demonstrates similar strengths to the blend sign in predicting HE, with a high specificity but relatively unreliable sensitivity and AUC when used alone. May be useful in combination with other imaging features.
Zhou et al. 2021 (19)	Meta-analysis 9 2,939 N/A	Original studies on the association between the island sign and HE in ICH Duplication, non-related titles and abstracts, no clear time of initial CT scan, or no mention of the island sign	Pooled sensitivity, specificity, and SROC curve of predicting HE by: Island sign for HE prediction	Standard NCCT; island sign	Findings suggest that the island sign in NCCT has valuable predictive accuracy for HE in sICH. Sensitivity, specificity, and AUC for the island sign for HE prediction were 50, 89%, and 0.73, respectively.	The island sign demonstrates improved sensitivity compared to the blend sign or black hole sign, with a minor decrease in specificity but significantly lower AUC in predictive power comparatively.
Yang et al. 2020 (20)	Meta-analysis 5 1,493 up to 2010	Original studies on the association between the satellite sign and HE in ICH Duplication, non-related titles and abstracts, no clear time of initial CT scan, or no mention of the satellite sign	Effect values (sensitivity, specificity, positive and negative likelihood ratios) for predicting HE by satellite sign use	Standard NCCT; satellite sign	Pooled diagnostic sensitivity and specificity were 0.50 and 0.71, respectively. AUC was 0.66. The satellite sign exhibits moderate use in predicting HE in patients with sICH.	The satellite sign demonstrates a relatively weak ability to predict HE in regards to specificity and AUC, compared to island, black hole, and blend signs.
Xu et al. 2018 (21)	Meta-analysis 29 5,514 1995–2017	Original studies reporting on the relationship between CTA spot sign and HE, mortality, or poor outcomes Duplication, non-English studies, non-related titles and abstracts, no clear time of initial CTA scan, or no mention of the spot sign	Effect values (sensitivity, specificity) on HE. Mortality (in-hospital and 3-month), poor outcomes (mRS at discharge and 3-months out)	CT angiography; spot sign	The incidence of CTA spot signs in sICH is relatively common (23.4%), with its presence demonstrating significantly higher risk of HE (OR 8.49), with a sensitivity, specificity, and AUC of 62, 88%, and 0.86, respectively.	CTA spot signs are relatively accurate predictors of HE, mortality, and poor outcomes post-discharge. Compared with standard NCCT signs, the spot sign provides a good diagnostic tool to predict HE and its related morbidity and mortality risks.
Wang et al. 2021 (22)	Prospective study 1 50 2014–2016	>18 years old, within 6 h of onset, NCCT+CTA + CTP at baseline, NCCT re-examination within 24 h of onset, and informed consent Patients without confirmed ICH, CT imaging performed >6 h after symptom onset, lack of CT imaging at 24 h mark, death within 24 h, or hematoma evacuation within 24 h	HE incidence (>33% from baseline imaging)	CT perfusion; cerebral blood volume/flow	Ipsilateral peri-edema CBF may be an independent risk factor for early HE (OR 1.095, *p* = 0.024), with a mild but significant prediction value of higher CBF for early HE after sICH.	Higher CBF may mildly predict early HE after sICH. CBV, on the other hand, demonstrates an inverse relationship with HE (66), independent of ICH volume. Further studies are needed to determine the relationship between CBV and CBF on predicting HE for clinical utility.

AUC, area under curve; CBF, cerebral blood flow; CBV, cerebral blood volume; CT, computed tomography; CTA, computed tomography angiography; CTP, computed tomography perfusion; FAST, Factor VII for Acute Hemorrhagic Stroke Treatment; GCS, Glasgow Coma Scale; HE, hematoma expansion; ICH, intracerebral hemorrhage; OR, odds ratio; mRS, modified Rankin Scale; NCCT, non-contrast computed tomography; RCT, randomized clinical trial; sICH, spontaneous intracerebral hemorrhage; SROC, summary receiver operating characteristic curve.

**Table 2:** Summary of clinical trial data and pilot analyses evaluating radiomics in predicting hematoma expansion in spontaneous intracerebral hemorrhage.

Study authors, date	(1) Study design (2) N studies (3) N patients (4) years studied	(1) Inclusion criteria; (2) exclusion criteria	(1) Primary outcome; (2) secondary outcome	Imaging modality and radiology signs	Main study findings	Limitations and interpretation
Haider et al. 2023 (23)	*Post hoc* analysis of ATACH-2 trial 1 897 N/A	All ATACH-2 trial participants CT artifacts, corrupted images, or missing CT images; missing clinical data if adequate CT	HE prediction (AUC for discovery and independent validation cohorts) Functional outcomes at 3-months post-treatment	Modified NCCT images; model with various signature combinations of select radiomic, clinical, and visual markers	The utilization of a combination of radiomic features and clinical variables provides the highest predictive power of HE after sICH (AUC 0.67, 0.64), outperforming clinical (*p* = 0.02) and visual signatures (*p* = 0.03), and a combined visual/time (BAT) score (*p* < 0.001).	Radiomic features on admission NCCT appear to predict HE with stable generalizability. The combination of radiomic and clinical predictors may provide the highest predictive value for clinicians in immediate and long-term predictions of HE and their associated morbidity and mortality.
Li et al. 2023 (24)	Prospective 1 214 2011–2017	Ages 18–80 years, spontaneous ICH, baseline CT scan within 6 h of symptom onset, follow-up CT within 36 h of admission CT scan Any patients with known or suspected secondary causes of sICH	Receiver operating characteristic analysis and AUC of HE predictive value	Modified NCCT images; model with various signature combinations of select radiomic, clinical, and visual markers	Of the 8 machine learning methods selected for constructing models, the MLP model was demonstrated to be the best model for HE prediction after sICH. Sensitivity, specificity, and AUC in MLP models were 90.0, 87.9%, and 0.92, respectively.	This study demonstrated how NCCT models based on radiomics features and machine learning, particularly the MLP model, can greatly enhance clinicians in predicting early perihematomal edema expansion after sICH. Such high specificity, sensitivity, and AUC results have not been seen in standard manual models together in this review.
Dai et al. 2023 (25)	Retrospective 1 187 2017–2022	First CT scan within 24 h and second within 24 h of first scan, >18 years of age, history of hypertension, complete clinical laboratory tests Long-term anticoagulant use, known or suspected secondary causes of sICH, hemorrhage after infarction, primary intraventricular hemorrhage, or poor image quality	HE risk and occurrence (33% or > 6 mL increase in hematoma volume)	Modified NCCT images; nomogram model	13 radiomics features were selected to construct this group’s radiomics signature, which demonstrated a robust ability and high accuracy to predict HE after hypertensive ICH. ROC models demonstrated AUC up to 0.89 and 0.82 in training and validation sets. Predictive capacity increased further to 0.90 and 0.88 once their model included the blend sign.	The combination of prediction models of radiomics with the blend sign, commonly utilized in manual image interpretation, demonstrates high prediction performance, similar to the MLP model seen in the Li et al. study (76). This introduces the idea of trialing various radiomic models with various image features or signs to help increase predictive potential for HE risk.
Feng et al. 2023 (26)	Retrospective 1 561 N/A	Patients presenting with sICH and baseline NCCTs	Intersection over union, dice coefficient, and accuracy in HE detection	Modified NCCT images; nomogram model	This study utilized deep learning to automate the segmentation of the ROI before performing a radiomics analysis to predict HE. When done in a fully automatic fashion, this combined model provides relatively high (AUC 0.87 and 0.82) predictive potential in HE risk judgement.	This is an additional study to demonstrate the relative accuracy and predictive potential a combined model can have on efficiently predicting early HE, with AUCs rivaling those of manual detection of spot signs clinically.
Rezaei et al. 2023 (27)	Retrospective 1 116 2004–2019	Patients >18 years old with sBGH on brain CT at time of admission and follow-up brain CT within 24 h of admission Patients <18 yearsor missing necessary CT imaging or lack of sBGH findings on admission	Performance to predict HE via AUC	Modified NCCT images; 10 different machine learning models utilized	This study used 10 different machine learning models developed based on clinical data (demographics, labs, exam), radiology signs (discussed in this table), and radiomic features. AUC of 0.9 and 0.89 were demonstrated in predicting HE.	The AUC for these models based on 10 select radiomic features appear to provide the best accuracy in predicting HE, regardless of depth of sICH, as these patients all had sBGH, compared with the other studies often being lobar sICH. This shows the accuracy for any intracranial bleed at any size or depth, while still being better than many of the clinical and standard radiology features utilized in manual reads.
Seymour et al. 2022 (28)	Retrospective 1 200 2016–2019	Patients presenting with stroke-like symptoms with a baseline NCCT and T2 MRI, with follow up NCCT scans	HE (>33% or > 6 mL from baseline imaging) prediction potential via AUC	Standard NCCT and T2 MRI; 7 different machine learning models utilized	This study found tht NCCT models were better predictors of HE than MR models (sensitivity, specificity, and AUC of 73, 71%, 0.70 vs. 72, 67%, 0.69), with logistic regression classifiers best to use in NCCT radiomic models.	Radiomic models shown to be accurate and highly useful in either CT or MRI-based imaging. Given the similarities in predictive potential, CT may be preferred given its rapid abilities in such acute pathologies.

AUC, area under curve; CT, computed tomography; HE, hematoma expansion; MLP, multilayer perceptron; MRI, magnetic resonance imaging; NCCT, non-contrast computed tomography; ROC, receiver operating characteristic curve; ROI, region of interest; sBGH, spontaneous basal ganglia hemorrhage; sICH, spontaneous intracerebral hemorrhage.

## 3 Hematoma expansion

### 3.1 Pathophysiology and natural history

The definition and pathophysiology of HE are nuanced because the line between primary and secondary bleeding can be difficult to delineate. Dowlatchahi et al. (29) outlined common definitions of HE as used in clinical trials based on absolute and/or relative volume changes on serial CT imaging, including a 40% relative volume increase or an absolute volume increase of 12.6 mL, a 33% relative volume increase, a 20 mL absolute volume increase, or a combined 50% relative volume increase and a 2 mL absolute volume increase. Notably, these authors demonstrated that no matter the threshold for the definition of HE, each was independently related to poor outcomes (29). The cause of continued bleeding, or HE, has garnered two prominent schools of thought: further hemorrhage from the primary leaky vessel or the “snowball” effect, which infers continued hemorrhage from smaller neighboring vessels (30, 31).

Several studies have illustrated the impact of HE on morbidity and mortality in patients with sICH. In a retrospective study by Davis et al. (32), each 10% increase in hematoma volume was associated with a 5% increase in mortality rate. Similarly, as part of the Intensive Blood Pressure Reduction in Acute Cerebral Hemorrhage Trial (INTERACT1) randomized controlled trial (RCT), Delcourt et al. (33) found that a 1 mL increase in hematoma volume leads to a 5% greater risk of death or functional dependency. A report by Edlow et al. (34) presented a particularly illustrative case of HE in which a patient experienced an acute sICH while in the MRI scanner; what began as an 8.8 mL hemorrhage grew to 20.5 mL within 10 min and eventually settled at 43.5 mL just over 22 h after the initial finding. Although the patient initially presented with mild neurologic deficits, a new left-sided hemineglect was noted directly after the imaging-documented HE. Recent work by Morotti et al. (35) has further defined those patients at highest risk for a poor outcome with HE as those with a smaller initial HE volume (<30 mL), with either an absolute HE increase of >12.5 mL or a relative increase in HE volume of >66%. These reports highlight the detrimental short- and long-term impact of HE on patient outcomes and the potential utility of identifying patients at the highest risk for HE after sICH who may benefit from stepwise implementation of aggressive preventative treatments.

### 3.2 Treatment adaptations

Iterative treatment guidelines exist for patients with sICH, with the most recent version published in 2022 (36). Cornerstones of management include acute blood pressure control, anticoagulant reversal, and consideration of either minimally invasive surgical evacuation or open craniotomy/craniectomy evacuation in selected patients, with acute neurological deterioration from HE often accelerating the treatment algorithm (36). Given its association with poor outcomes, significant effort has been devoted to limiting HE after sICH.

Multiple RCTs have examined the role of intensive blood pressure reduction in preventing HE and mortality and improving outcomes after sICH, including the Intensive Blood Pressure Reduction in Acute Cerebral Hemorrhage Trial 2 (INTERACT2), followed more recently by the INTERACT3 and INTERACT4 trials, and the Antihypertensive Treatment of Acute Cerebral Hemorrhage II (ATACH-2) trial (37–40). The INTERACT2 study published in 2013 included 2,794 patients who had sICH within the previous 6 h and had elevated blood pressure; these patients were randomly assigned to a control arm (target systolic level < 180 mm Hg) or a treatment arm that involved intensive blood-pressure lowering to a systolic blood pressure of less than 140 mm Hg within 1 h of randomization and maintained for 7 days (37). Similarly, the ATACH-2 trial published in 2016 included 1,000 sICH patients with hypertension who were randomly assigned to either a control (goal systolic blood pressure of 140 to 179 mm Hg) or a treatment (goal systolic blood pressure of 110 to 139 mm Hg) arm within 4.5 h of symptom onset and continued for the 24 h (38). In both trials, despite prior evidence of the potential efficacy of rapid blood pressure control in limiting HE (41), there was no significant reduction of the primary outcome of morbidity and mortality or the secondary outcome of HE across treatment groups (37, 38). Secondary analyses of these data have nonetheless supported a beneficial role for systolic blood pressure control, including a post-hoc analysis of the INTERACT2 trial that linked elevated blood pressure within 24 h post-bleed to increased rates of death and disability at 90 days (42). Moreover, in a post-hoc analysis of the ATACH-2 data by Li et al. (43), a subgroup of patients that received intensive blood pressure control within 2 hours of symptom onset was found to have a significant reduction in the risk of HE (odds ratio [OR], 0.56; 95% confidence interval [CI], 0.34–0.92; *p* = 0.02) and improved functional outcomes, suggesting a time-sensitive treatment window for hypertension management. The 2022 guidelines summarize these findings and suggest acutely targeting a systolic goal of 130–150 mm Hg in most patients with sICH and hypertension (36).

The INTERACT3 and 4 trials were published in 2023 and 2024, respectively, and assessed whether bundled protocols in addition to systolic blood pressure control or pre-hospital blood pressure reductions prior to a definitive diagnosis could improve sICH outcomes (39, 40). In INTERACT3, 7,036 adult patients who presented with sICH within 6 h of onset were randomized, with 3,221 patients assigned to a goal-directed care bundle that combined early intensive blood pressure lowering (systolic <140 mm Hg) with management algorithms for correction of hyperglycemia, pyrexia, and abnormal anticoagulation (all within 1 h) and 3,815 patients assigned to a “usual care” group based on local treatment standards (39). Although HE was not assessed in this study, the primary outcome of functional recovery at 6 months (as measured by modified Rankin Scale [mRS] score) was significantly more likely in the care bundle group (OR 0.86; 95% CI 0.76–0.97; *p* = 0.015). INTERACT4 assessed whether blood pressure control in undifferentiated stroke patients prior to arrival at the hospital could improve outcomes (40). In this work, 2,404 patients with a suspected acute stroke (that caused a motor deficit) who had an elevated systolic blood pressure ≥ 150 mm Hg and were assessed in an ambulance within 2 h of symptom onset were randomized to receive immediate systolic blood pressure control (target 130–140 mm Hg) (1,205 patients) or “usual” blood pressure management commencing after hospital arrival (1,199 patients). Although again HE was not a focus of this study, blood pressure treatment before hospital arrival did not affect the primary outcome of functional status at 90 days (based on mRS) (OR, 1.00; 95% CI, 0.87 to 1.15) (40). However, this was the result of including a cohort with mixed underlying pathologies: patients with ischemic stroke were more likely to have a poor functional outcome with pre-hospital blood pressure control (OR, 1.30; 95% CI, 1.06 to 1.60), whereas patients with ICH were less likely to have a poor functional outcome (OR, 0.75; 95% CI, 0.60 to 0.92). Together, these studies support the importance of an expedited diagnosis and early, intensive protocolized medical management of patients with sICH.

Expedited surgical intervention may also play a role in the prevention of HE. Currently, surgical indications for evacuation of ICH vary with presentation. The authors of a 2020 systematic review identified five RCTs that focused on the optimal surgical management of sICH (44). Two of the largest studies at the time (Surgical Trial in Lobar Intracerebral Hemorrhage [STICH] 1 and 2) compiled a total of 1,634 patients. They failed to show clinical improvement in terms of the Glasgow Outcome Scale, Barthel Index, or mRS scores with open surgical evacuation of cortical sICHs versus medical management (44–46), despite a post-hoc analysis of STICH 1, suggesting that patients with hematomas <1 cm from the cortex may benefit from surgery. The other three smaller trials examined a total of 719 patients with subcortical sICH treated with minimally invasive (stereotactic and endoscopic) techniques versus medical management. They reported potential benefits of surgery as indicated by decreased mortality rates and improved functional outcomes using the mRS and Barthel Index (44, 47–49). A subsequent RCT (Minimally Invasive Surgery Plus Alteplase for Intracerebral Hemorrhage Evacuation [MISTIE III]) explored the role of minimally invasive sICH evacuation combined with direct thrombolysis in 505 patients. Although it did not show a significant improvement in its primary functional endpoint (i.e., mRS 0–3 at 1 year), mortality was significantly lower with surgery, and an increased rate of good functional outcomes was seen with a clot reduction to <15 mL on secondary analysis (50). Building on this, the recently completed Early Minimally Invasive Removal of Intracerebral Hemorrhage (ENRICH) trial was designed to evaluate the role of minimally invasive trans-sulcal parafascicular surgery for sICH (36); its data revealed significantly improved clinical outcomes (as determined by utility-weighted mRS at 180 days) with surgery performed within 24 h for lobar hemorrhages between 30 and 80 mL volumes (51). Although they did not include the recent ENRICH data, which solidify the role of surgery in these patients, the current 2022 sICH guidelines suggest that minimally invasive surgery be considered for patients with supratentorial ICH of greater than 20 to 30 mL volume with GCS scores of 5–12, with or without thrombolytic use (36). Although the potential for HE does not currently impact the decision for surgery in sICH, HE can change a nonsurgical candidate to a surgical candidate. As surgical techniques continue to improve and become less invasive, identifying consistent markers of HE may allow for more aggressive upfront surgical intervention in patients at risk, thereby avoiding the functional decline commonly associated with HE.

## 4 Standard imaging features of HE

Given the ubiquity of standard imaging modalities in the diagnosis and management of sICH, significant efforts have been made to identify predictive markers of HE after sICH on non-contrast CT, CT angiography, and CT perfusion (Table 1). This is analogous to other standard radiologic reviews, wherein common characteristics of known pathologies can alter clinical care significantly. For example, close interpretation of conventional MRI sequences can often differentiate between low- and high-grade intrinsic brain tumors based on patterns of enhancement, edema, necrosis, and multifocality, with an added ability to suggest tumor subtype based on location and secondary features such as calcifications and appearance on diffusion imaging (52). Although these efforts have demonstrated promise with sICH, in general, such techniques have not achieved the high levels of sensitivity and specificity required to confidently alter clinical care regarding HE. Likely undermining their clinical efficacy is a heavy reliance on subjective interpretations and potentially vague or overlapping characteristics (53). A brief review of some of the most described imaging markers are provided below.

### 4.1 Location

Hemorrhage locations are often defined as lobar (cortex and cortical–subcortical junction) or deep (thalamus, basal ganglia, internal capsule, and deep periventricular white matter), and while debate exists, differences in propensity for HE have been suggested based on location (54). For example, in a recent post-hoc analysis of 728 patients from the FAST (Factor VII for Acute Hemorrhagic Stroke Treatment) trial for supratentorial hemorrhage, lobar hemorrhages were found to be larger at baseline (35 vs. 12 mL, *p* < 0.001) and had a higher risk of HE (44 vs. 27%, *p* = 0.001) than deep hemorrhages (14). However, this differs from other studies in which deep hemorrhages were found to have a higher risk of HE (55, 56), highlighting the poor prognostic potential of sICH location for HE.

### 4.2 Initial size

The size of the hematoma at presentation has likewise been suggested as a marker for HE, with bigger initial ICH size linked to a higher HE risk (15). Brouwers et al. (15) included the initial size of the ICH (<30, 30–60, and > 60 mL) as part of their 9-point prediction score, which was independently validated to help predict HE in sICH (C statistics score of 0.72 for the development cohort and 0.77 for the independent validation cohort). In their multicenter prospective analysis, Silva et al. (57) reported that among 183 patients who presented for sICH management, 22.3% of patients with sICH volumes <20 mL but 33.6% of patients with sICH volumes ≥20 mL experienced HE. Similarly, Dowlatshahi et al. (58) demonstrated that smaller hematomas, particularly those under 10 mL, are less likely to expand, leading to better neurological outcomes. Although these data provide additional justification to the current recommendations to consider surgery in selected patients with supratentorial sICH volumes >20 mL based on an increased HE risk (36), volume alone is unlikely to be a reliable marker of HE risk.

### 4.3 Shape

Irregular sICH shape has also been linked to a greater risk of HE in multiple studies (16, 59, 60), and is thought to result from multifocal hemorrhage rather than a singular hemorrhage source. This fits with an analysis of the INTERACT2 trial data by Delcourt et al. (61), wherein irregular sICH shape independently correlated with death and major disability at 90 days post-sICH (OR, 1.60; 95% CI, 1.29–1.98) as well as a lower functional outcome as determined by mRS score (OR, 1.46, 95% CI; 1.23–1.72). A meta-analysis of data from more than 2000 patients on irregular shape on non-contrast CT as a predictor of HE after sICH nonetheless demonstrated an overall sensitivity, specificity, and an area under the receiver operating characteristic curve (AUC) of 67, 47%, and 0.61, respectively (16).

### 4.4 Blend sign

The blend sign refers to a blending of adjacent hypoattenuating and hyperattenuating regions within a hematoma on non-contrast CT (defined by a difference of 18 Hounsfield units between densities) (62, 63) and correlates with the presence of a spot sign (a marker of ongoing hemorrhage) on CT angiography (63). In a retrospective analysis of 238 patients, Sporns et al. (63) linked the blend sign to a more than threefold increase in both risk of HE and a poor neurologic outcome after sICH. In a meta-analysis examining 2,248 patients, however, Yu et al. (17) found that the sensitivity, specificity, and AUC of the blend sign for HE were 28, 92%, and 0.85, respectively.

### 4.5 Black hole sign

The black hole sign is characterized as a well-defined hypodense area within a hyperdense hematoma on non-contrast CT and differs in criteria from the blend sign in that there must be a 28-Hounsfield unit difference between the hypodense and hyperdense regions (64, 65). Similar to the blend sign, it is correlated to the spot sign on CT angiography (a marker of ongoing hemorrhage). Although it has also been suggested as a marker of HE (64, 65), a meta-analysis of the predictive value of the black hole sign in more than 1,495 patients reported a sensitivity, specificity, and AUC of this sign of 30, 91%, and 0.78, respectively (18).

### 4.6 Island sign

The island sign refers to sICH margin irregularities on non-contrast CT (66) and is defined as either ≥3 minor hematoma regions that do not visibly communicate with the primary hematoma or ≥ 4 minor hematomas that lobulate separately from the main hematoma (66, 67). It is thought to result from secondary multifocal areas of hemorrhage that result from the main hematoma and is linked to HE and poor outcome after sICH (66). In a meta-analysis of 2,939 patients, the sensitivity, specificity, and AUC of the island sign as a predictor of HE were 50, 89%, and 0.73, respectively (19).

### 4.7 Satellite sign

The satellite signal refers to the presence of small high-density starry dots near but distinct from the primary hematoma on non-contrast CT, with a diameter of ≤10 mm and a distance of ≤20 mm from the main hematoma body (67, 68). They are thought to represent small foci of secondary reactive hemorrhage and have been linked to HE and worse functional outcomes after sICH (67, 68). A meta-analysis of 1,493 patients nonetheless reported a sensitivity, specificity, and AUC of the satellite sign as a predictor of HE of 50, 71%, and 0.66, respectively (20).

### 4.8 Spot sign

Expanding beyond non-contrast CT, the spot sign has been suggested as a marker of continual intracranial bleeding after sICH as visualized on CT angiography (69). It is generally considered to be a more reliable marker of HE than non-contrast CT-based signs and has been associated with worse functional outcomes after sICH (70, 71). In a broad systematic review and meta-analysis comprising 29 studies and 5,514 patients, Xu et al. (21) reported a spot sign was identified in 23.4% of sICH patients and was associated with a significantly greater risk of HE (OR 8.49, 95% CI 7.28–9.90). In support of its likely better predictive value over non-contrast CT signs, the sensitivity, specificity, and AUC of the spot sign as a marker of HE in this work were 62, 88%, and 0.86, respectively (21). The spot sign on MRI studies has also been explored in a recent study, with a similar association with HE and poor outcome after sICH (72).

### 4.9 Cerebral blood volume and flow

Perihematomal cerebral blood volume, as determined by CT perfusion, has also been linked to HE (73), with previous hypotheses suggesting that bleeding will continue without adequate counter-pressure from blood-saturated perihematomal parenchyma. In support of this concept, in a small study of 155 patients, Morroti et al. (73) recently demonstrated that perihematomal cerebral blood volume (independent from blood pressure, hematoma volume, and other potential confounders) was inversely associated with HE (B = −0.20; *p* < 0.001), with only very low cerebral blood volumes of <1.4 mL/100 g being associated with HE (B = 0.25, *p* < 0.001). This nonetheless differs from a more recent small study of 50 patients with sICH, in which higher cerebral blood flow around the hematoma on CT perfusion imaging was positively correlated with incidence of HE (*p* = 0.004) (22).

## 5 The potential utility of radiomics

Although a multitude of standard imaging and combined standard imaging and clinical criteria have been suggested as markers of HE (15, 17, 18, 20, 21, 74), their practical use has largely not caught on in sICH management, potentially because of a combination of low specificity, difficulty in radiologic interpretation, or cumbersome scales. Differing from the above techniques, radiomic analysis extends beyond the limits of human intuition, ability, and knowledge by integrating pathology-specific imaging characteristics and patterns with a granularity well beyond the human eye, with practical implications for predicting HE (Table 2). Van Timmerman et al. (75) detailed the general process of radiomic analysis as follows: (1) image acquisition, (2) image segmentation to define the regions of interest [ROIs; done manually, semi-automatically, or fully automatically (i.e., deep learning algorithms)], (3) image processing (to homogenize images based on pixel spacing, grey-level intensity, etc.), (4) feature extraction (commonly based on intensity, shape, texture, and radial features), and (5) feature selection/dimension reduction (identifying and optimizing the relevant features for statistical and machine-learning/artificial intelligence modeling).

Multiple technical considerations arise when implementing radiomic analyses into a heterogeneous healthcare environment, particularly variance and artifact in source imaging. Radiomics analyses of sICH typically use non-contrast CT scans. To reduce variance, scans from a single scanner may be used. However, techniques for image homogenization can allow pooling of images from different scanners. Images are then aligned and resampled using image processing software. Supervised quality checks are commonly performed to exclude images with significant artifact. Skull stripping (removal of non-brain tissue signal) is often performed as part of imaging processing and is particularly important for sICH located adjacent to the skull.

For the next step of image segmentation, ROIs may be selected using a color scale based on Hounsfield units. They can be segmented manually, wherein an experienced user traces the hematoma on each slice of the image, ensuring that the entire hematoma is included while excluding surrounding cerebral edema. However, some groups also use semi- and fully automated pipelines to select ROIs before performing a radiomics analysis (76). After segmentation is complete, shape, first-order (based on single-pixel or single-voxel analyses), and texture features are extracted from the images.

For machine learning-based radiomics models as part of the last step of feature selection/dimension reduction (77), cases are randomly divided into training and test sets. Extracted features are processed and normalized, and then subsamples of the training data are used for cross-validation to build and evaluate the classification model. Signatures can also be generated by combining radiomics features with visual markers (as determined by expert human reviewers, commonly neuroradiologists) and clinical variables into a least absolute shrinkage and selection operator-regularized logistic regression (LASSO-LR) model, among other methods (23). Model efficacy is determined by the mean AUC of the receiver operating characteristic curve, sensitivity, specificity, and accuracy. These parameters can be used to make statistical comparisons between models and identify the most predictive signature. Notably, the predictive efficacy of radiomic assessments can vary widely based on the machine-learning algorithm utilized (e.g., logistic regression, support vector machine, k-nearest neighbor, multilayer perceptron, random forest) (24), highlighting the importance of transparency in the reporting of these studies to ensure reproducibility, as well as the benefit of testing multiple algorithms to ensure an optimized model.

To date, most radiomic algorithms have been deployed in the oncologic setting. However, pilot analyses demonstrating the utility of a radiomics approach for pathology characterization have also been used in other intracranial applications ranging from classically non-descript temporal lobe tumors to MRI-negative epileptogenic mesial sclerosis (78, 79). To assess the hypothesis of HE detection via radiomics methodologies, Haider et al. (23, 38) recently examined the prospective ATACH-2 patient database of 897 total patients for markers of HE on non-contrast CT using discovery and independent validation cohorts. Their radiomics model (after manual segmentation of the sICH) included 1,130 total imaging features (18 first-order, 14 based on shape, and 75 based on texture on the original images and 11 derivative images) and resulted in an AUC for predicting HE of 0.64 (0.59–0.70) in the discovery cohort and 0.61 (0.56–0.67) in the independent validation cohort, respectively (23). Each of the markers was also significantly correlated with functional outcomes at 3-month follow-up (23). Additionally, their model compared favorably with visually dependent interpretations by three expert readers looking for eight reported HE markers on non-contrast CT (including the blend sign, swirl sign, black hole sign, island sign, satellite sign, and irregular shape) (23). Suggesting a role for the importance of multifactorial inputs, a radiomics signature combined with a clinical signature (incorporating only the baseline National Institutes of Health Stroke Scale [NIHSS] score) attained the highest AUC scores in this work (0.67 in the discovery cohort and 0.64 in the independent validation cohort) (23). This, notably, did not include the addition of standard non-contrast CT signs, which did not augment the predictive capacity of radiomics when added into the model in this work.

Similar recent studies have reported even higher predictive values for HE after sICH in optimized machine-learning models. In one study of 214 patients, Li et al. (24) found a sensitivity, specificity, and AUC of 90.0, 87.9%, and 0.92, respectively, using a logistic regression machine-learning algorithm. This model also involved manual segmentation followed by automated radiomics feature extraction based on geometry, intensity, and texture to generate 1906 features per patient, with such granularity facilitating a significantly higher predictive capacity than any of the human-detected signs discussed previously. A similar recent study of 187 patients by Dai et al. (25) supports the high predictive ability of radiomics for HE, with AUCs of 0.9 and 0.82 in the training and validation testing, respectively. Interestingly, unlike in the prior work by Haider et al. (23), the addition of a blend sign to the radiomics modeling in this study increased the predictive capacity, resulting in AUCs in training and validation testing 0.90 and 0.88, respectively (25). Taking this one process step further, a study of 561 patients by Feng et al. (26) used deep learning to automate segmentation of the ROI before performing a radiomics analysis to predict HE. The predictive value of radiomics in this fully automated model remained high, with AUCs in a training cohort of 0.87 and 0.82 in an external validation cohort (26).

Although many of the above studies did not specify lobar versus deep sICH location, the potential of radiomic analyses in sICH is likely unaffected by hemorrhage location. Rezaei et al. (27) examined HE in 116 patients with basal ganglia sICH. Their machine-learning algorithm incorporated 10 predictive noncontrast CT radiomics features and achieved an AUC of 0.9 on the training cohort and 0.89 on the testing group, significantly better than the AUCs ranging from 0.5 to 0.6 using clinical laboratory and standard radiology features (27). Although their study was not focused on HE, Wang et al. (80) demonstrated the potential of radiomics in infratentorial sICH by using a deep-learning model to predict the prognosis of primary pontine hemorrhages, with AUCs of 0.886, 0.886, and 0.759 in predicting 30-day mortality, 90-day mortality, and 90-day functional outcome. With the addition of clinical factors to their model, the predictive capacity improved these AUCs to 0.920, 0.941, and 0.894, respectively (80). Radiomic models using MRI as a primary imaging modality have also been explored as markers of HE after sICH. In a small comparative analysis of CT- versus MRI-based radiomics modeling, MRI demonstrated a similar optimized sensitivity, specificity, and AUC (up to 72, 67%, and 0.69, respectively) to non-contrast CT (73, 71%, and 0.70, respectively), although the reported predictive capacity for HE was lower than that seen in other studies across modalities (28).

Although these reports are preliminary, they support the potential of radiomics and deep learning modeling in sICH management and HE prediction. Rather than relying on subjective and often ambiguous imaging markers, radiomic analysis allows for objective integration of image volumes, voxel density, and textures unseen to the human eye that, when combined with deep learning modeling and fully automated workflows, can produce superior and clinically relevant predictive results. Although the role for integrating clinical (age, NIHSS, etc.) or standard imaging (spot sign, etc.) data in radiomic modeling of HE remains unclear, a fully automated workflow that is capable of highly accurate predictions based on non-contrast CT remains the working goal of this field.

## 6 Limitations and future directions

Given the ongoing pace of technological advances within medicine and beyond, optimized radiomics analyses—despite their relative infancy—are likely to play a future role in the clinical management of sICH, in addition to other cause of ICH such as cavernous malformations (81) and arteriovenous malformations (82, 83). However, inherent limitations of radiomics are applicable, including limitations of data sharing that can limit training model size, reproducibility, and optimization and, once clinical integration becomes a reality, addressing potential disparities in technological access and a need for clinician education (84).

Despite such obstacles, there are current precedents for clinical integration of radiomics, including within the field of genomics, where radiomics techniques are used to more accurately predict hereditary profile characteristics and anticipate survival after radiotherapy of patients with non-small cell lung cancer and nasopharyngeal carcinoma (10). Commercially available software that uses radiomics to detect large-vessel occlusions for thrombectomy is also becoming widely used, including at our institution, to streamline the care of patients after stroke (85). A similar application of radiomics for HE prediction, if sufficiently accurate, has the potential to optimize care of patients at high risk for HE by enabling highly tailored medical and surgical decision-making with a goal of mitigating adverse events and enhancing outcomes.

## 7 Conclusion

Radiomics is an emerging tool that leverages machine-learning algorithms for a variety of clinical applications. Emerging data have supported its potential future role in prediction of HE after sICH, likely enabling tailored, risk-stratified care to optimize patient outcomes. Refinement in radiomics modeling and inputs and expansion of training and validation databases will be needed before clinical integration.

## Author contributions

ST: Data curation, Investigation, Writing – original draft, Writing – review & editing. MF: Data curation, Investigation, Writing – original draft, Writing – review & editing. KC: Data curation, Investigation, Writing – original draft, Writing – review & editing. DG: Data curation, Investigation, Writing – original draft, Writing – review & editing. JN: Data curation, Investigation, Writing – original draft, Writing – review & editing. JB: Data curation, Investigation, Writing – original draft, Writing – review & editing. CO: Data curation, Investigation, Writing – original draft, Writing – review & editing. MBo: Data curation, Investigation, Writing – original draft, Writing – review & editing. MBr: Data curation, Investigation, Writing – original draft, Writing – review & editing. WC: Writing – review & editing, Supervision, Project administration, Validation, Resources. RR: Conceptualization, Data curation, Investigation, Project administration, Resources, Supervision, Writing – original draft, Writing – review & editing.

## Footnotes

^1^ https://pubmed.ncbi.nlm.nih.gov
